# Eruption of Odontomas into the Oral Cavity: A Report of 2 Cases

**DOI:** 10.1155/2014/639173

**Published:** 2014-05-11

**Authors:** Sreenivasan Bhargavan Sarojini, Ektah Khosla, Thomas Varghese, Leena Johnson Arakkal

**Affiliations:** ^1^Department of Oral Pathology and Microbiology, Mar Baselios Dental College, Thankalam, Kothamagalam, Ernakulam, Kerala 686691, India; ^2^Department of Pedodontics, Mar Baselios Dental College, Thankalam, Kothamagalam, Ernakulam, Kerala 686691, India; ^3^Department of Oral Medicine and Radiology, Mar Baselios Dental College, Thankalam, Kothamagalam, Ernakulam, Kerala 686691, India

## Abstract

Odontomas are the commonest odontogenic tumors of the oral cavity and are by nature asymptomatic. They consist mainly of dental tissue that may or may not be arranged in an orderly fashion. Their presence is often detected accidentally or due to the presence of a dental disturbance such as an unerupted tooth. The very rarity of odontomas erupting into the oral cavity validates the need for more current literature on the phenomenon. Our report of two cases aims to present and discuss the rare event of an erupting odontoma with the dental community.

## 1. Introduction

The term odontoma was first coined by Paul Broca in 1867 and was employed as a generalized term to refer to all odontogenic tumors [1]. He defined them as “tumors formed by the overgrowth of transient or complete dental tissues” [2]. These lesions occur mostly within the bone though instances where it has been localized in the gingival have also been reported [3].

Recent times have seen this entity being regarded by some authors as more of a hamartoma than a true neoplasm [4]. According to the WHO classification (2005) odontomas are divided into 2 types: (a) compound odontoma—which consists of miniature tooth—like denticles, (b) complex odontoma—which consists of an irregular mass of hard and soft dental tissue [1, 5]. Another classification divides them into intraosseous and extraosseous odontomas based on their location [6].

They account for 22–67% of all maxillary tumors [7] with an increased prevalence in children and adolescents [4, 8]. Odontomas have a limited growth potential and are often detected on taking a routine radiographic examination [9] to determine the reason for an unerupted tooth or a retained primary tooth. Majority of all odontomas are detected during the first two decades of life [8]. The sex predilection is considered controversial as some authors report an equal distribution among sexes [10] while others report a male predominance [11] whereas yet another group have reported a female predominance [12]. In our case series one patient was male while the other was female.

The eruption of odontomas into the oral cavity is a rare phenomenon and may be associated with complications such as pain, infection, and delay in the eruption of permanent teeth. As of 2009 only 20 cases of erupted odontomas have been reported in the literature [7]. Though rare, dentists should be aware of the potential possibility of encountering such cases and the difficulties they pose with regard to treatment. The very rarity of odontomas erupting into the oral cavity validates the need for more current literature on this phenomenon.

## 2. Case Report

### 2.1. Case Report 1

**Figure 1:**
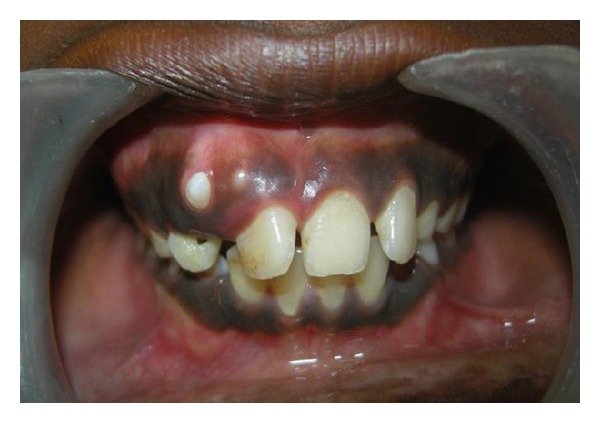
Clinical appearance of the erupting odontoma seen on the gingival between 11 and 12.

A 12-year-old male patient presented with complaint of the presence of a small hard mass in maxillary anterior region. He had no complaint of pain nor was any other relevant medical history elicited. The intraoral examination revealed a small whitish oval tooth-like structure present in maxillary anterior region between 11 and 12, and both teeth exhibited a slight rotation (Figure 1). On enquiry, the patient reported having noticed its presence since the age of nine. He reported no pain or any associated symptoms during or after the appearance of the lesion. No prior records or radiographs were available as this was the patient's first dental consultation.

**Figure 2:**
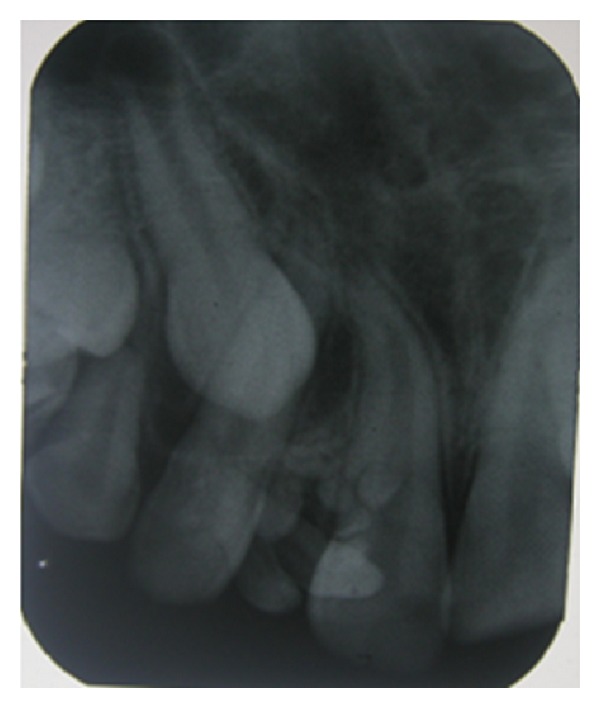
IOPAR showing radiographic appearance of odontoma.

**Figure 3:**
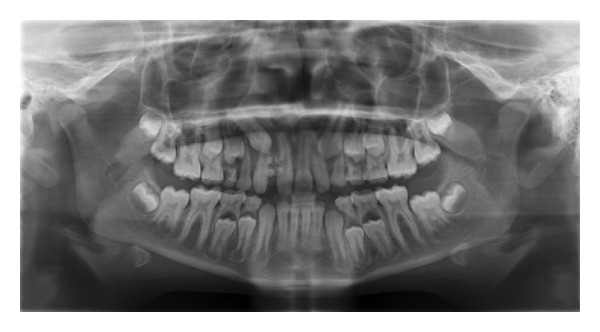
Panoramic view of the same patient.

The lesion was hard and nontender and the gingiva around it appeared normal. An intraoral periapical radiograph (Figure 2) and a panoramic radiograph (Figure 3) were taken which revealed irregular tooth-like structures similar to the density of dental tissues seen between 11 and 12 suggestive of an erupted compound odontoma.

**Figure 4:**
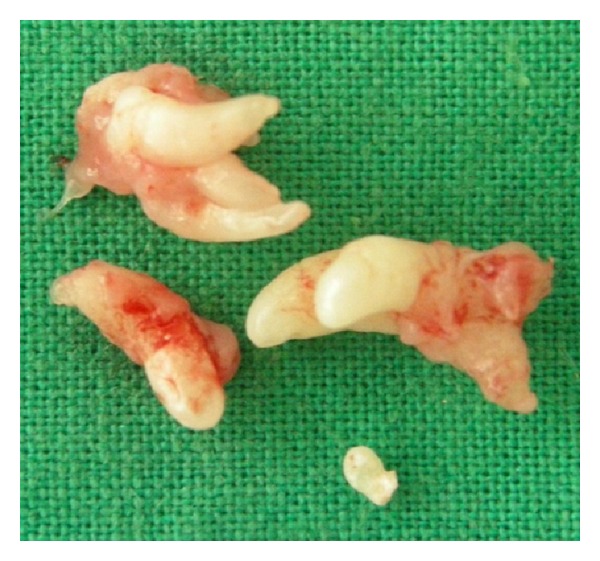
Specimen after surgical removal.

**Figure 5:**
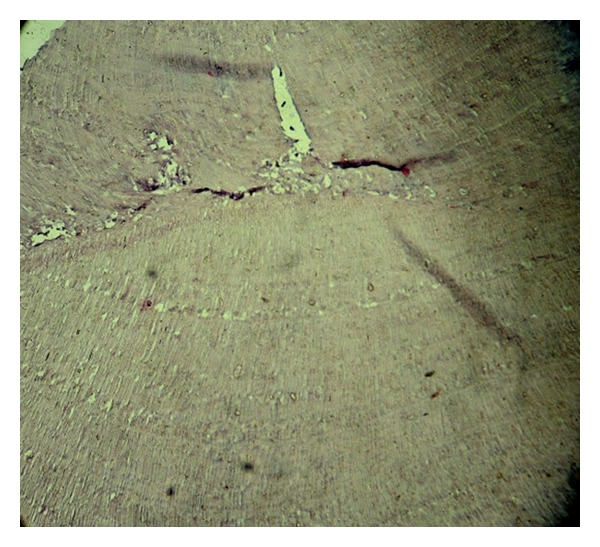
Histopathological section at 10x magnification.

The odontoma was removed surgically under local anaesthesia and the specimen (as seen in Figure 4) sent for histopathologic examination that revealed the specimen to be a compound odontoma with the microscopic appearance showing enamel, dentin, pulp chamber, and cementum resembling the structure of tooth as seen in Figure 5.

The patient was subsequently followed up for a period of one year and no recurrence of the lesion was seen.

### 2.2. Case Report 2

**Figure 6:**
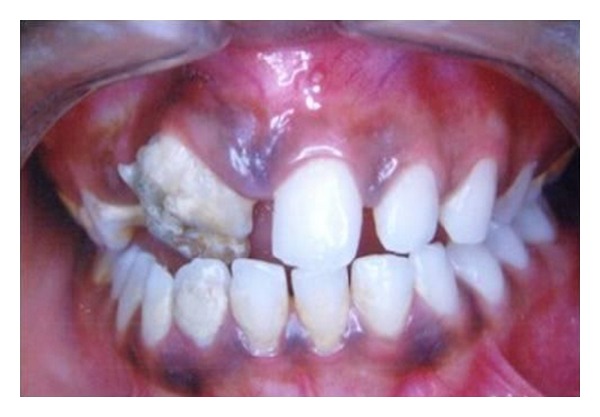
Intraoral view of erupting odontome in region of 11.

A 16-year-old female patient reported with the complaint of a mass in the anterior maxilla since the age of eight years. The eruption was not associated with pain or any other symptoms. During the intraoral examination an agglomerated mass of calcified structure was seen in the place of missing 11 and 12 (Figure 6).

**Figure 7:**
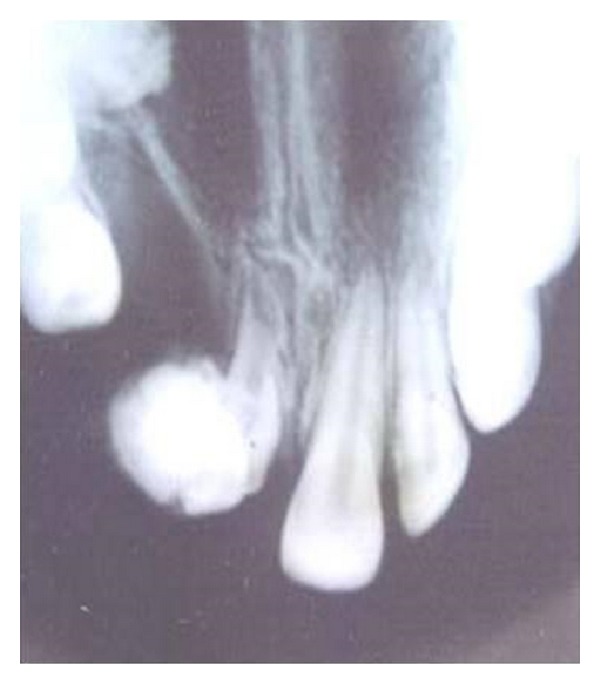
Maxillary occlusal view of the lesion.

**Figure 8:**
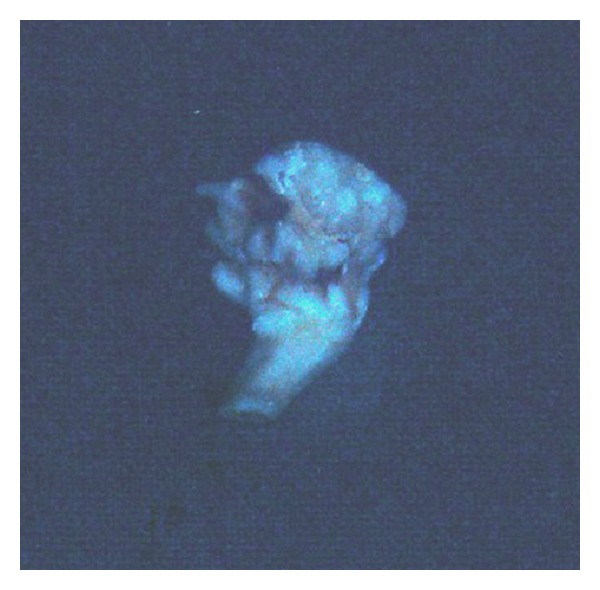
Specimen obtained after surgery.

**Figure 9:**
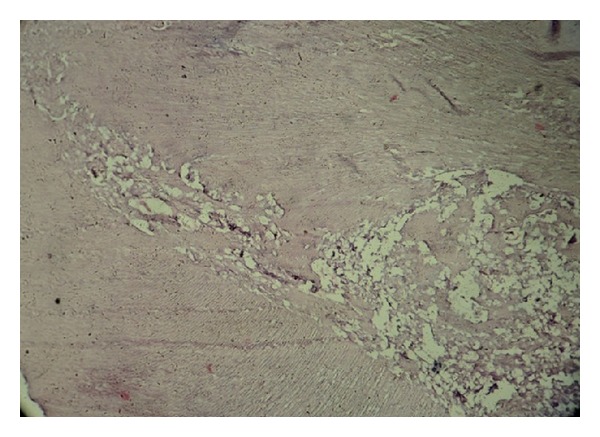
Histopathologic view of specimen under 10x magnification.

An anterior maxillary occlusal view showed a mass with varying radiodensity resembling enamel, dentin, and cementum. This mass appeared to be attached to a root-like structure with wide root canal and open root apex (Figure 7). The calcified mass was surgically removed (Figure 8) and sent for histopathologic examination which revealed the presence of enamel, dentin, pulp, and cementum as seen in Figure 9.

The patient is currently under follow-up for the last seven months and has not shown any incidence of recurrence so far.

## 3. Discussion

An odontoma is a tumor of unknown etiology and is found frequently associated with a dental germ in development, a supernumerary tooth, or even a retained primary tooth [9]. They are the most common type of tumor of odontogenic origin [13]. Of the two varieties in existence, the compound odontoma has shown a predilection for the anterior portion of the maxilla whereas complex odontomas show a preference for the posterior region of the mandible [14].

Their exact etiology remains obscure but possibly includes trauma to primary dentition, inflammatory and infectious processes, odontoblastic hyperactivity, mutant gene [15], and hereditary anomalies such as Gardner's syndrome and Hermann's syndrome [3]. Neither of our cases gave a history of any similar etiologies nor were they suffering from any syndromes. Odontomas are generally asymptomatic and rarely assumed dimensions greater than an average tooth, but if they attain an unusually large size can cause expansion of the cortical bone [16]. Both of our cases were asymptomatic and exhibited a size more or less within normal limits. The largest reported in the literature so far weighed 0.3 kg [17].

The spontaneous eruption of an odontoma into the oral cavity is an exceptional circumstance. Such an instance may be associated with pain, inflammation of the surrounding soft tissue, or infection and suppuration [18]. Due to the lack of periodontal ligament and therefore lack of contractility of fibroblasts around the odontoma, the mechanism of eruption is thought to be different from that of a conventional tooth. A few possibilities have been put forward and one of which states that the increasing size of the odontoma may lead to sequestration of the overlying bone and the subsequent occlusal movement. Another possibility implicates bony remodelling as a probable cause [5]. But despite the existence of such theories, the exact mechanism still remains unknown.

The radiographic appearance and histopathology are distinctive. The complex odontoma appears as an irregular calcified mass surrounded by a thin radiolucent area with a smooth periphery and the compound odontoma appears as multiple calcified tooth-like structures in the centre of a well-defined radiolucent region [19]. The histopathology most commonly reveals enamel matrix, dentine, pulp tissue, and cementum that may or may not exhibit a normal relationship surrounded by a connective tissue capsule that is similar to the normal dental follicle [20]. It is suggested that all odontomas be sent for a histopathological analysis in order to rule out ameloblastic odontoma and ameloblastic fibroodontoma and to obtain a definitive diagnosis [1, 12].

Timely detection and prompt surgical treatment followed by curettage are recommended to prevent complications such as tooth loss, cystic changes, bone expansion, and delayed eruption [19].

## 4. Conclusion

Odontomas are commonly occurring benign entities but rarely do they erupt into the oral cavity. We have presented two such rare cases where the odontomas have erupted into the oral cavity. Despite their benign nature, it is important that odontomas be treated promptly and conservatively so as to prevent the formation or facilitate the early correction of any dental and occlusal disturbances.
